# Informing creation of the FEEDS Toolkit to support parent-delivered interventions for eating, drinking and swallowing difficulties in young children with neurodisability: intervention use by neurodevelopmental diagnosis and healthcare professional role

**DOI:** 10.1136/bmjpo-2023-002394

**Published:** 2024-08-24

**Authors:** Emogene Shaw, Lindsay Pennington, Morag Andrew, Helen Taylor, Jill Cadwgan, Diane Sellers, Christopher Morris, Deborah Garland, Jeremy Parr

**Affiliations:** 1 Newcastle University Population Health Sciences Institute, Newcastle upon Tyne, UK; 2 Northumbria Healthcare NHS Foundation Trust, North Shields, UK; 3 Newcastle upon Tyne Hospitals NHS Foundation Trust, Great North Children's Hospital, Newcastle Upon Tyne, UK; 4 Paediatric Neurosciences, Evelina London Children's Hospital, London, UK; 5 Chailey Clinical Services, Sussex Community NHS Foundation Trust, Lewes, UK; 6 Peninsula Childhood Disability Research Unit (PenCRU), University of Exeter Institute of Health Research, Exeter, UK; 7 National Autistic Society, London, UK

**Keywords:** Health services research, Neurology, Growth

## Abstract

**Background:**

The FEEDS (Focus on Early Eating, Drinking and Swallowing) study focused on interventions used to improve feeding for children with neurodisability and eating, drinking and swallowing difficulties (EDSD), and the outcomes viewed as important by healthcare professionals (HPs) and parent carers. The FEEDS Toolkit was created subsequently as an intervention decision aid to be used collaboratively by parent carers and HPs. This study aimed to inform on current intervention practices and influence toolkit design by ascertaining whether specific intervention use varied by a child’s main diagnosis and by specific HP role.

**Methods:**

FEEDS survey data were grouped by child’s main diagnosis and HP role. Main diagnoses included autism spectrum disorder (ASD) n=183; Down syndrome (DS) n=69; cerebral palsy (CP) n=30). HPs included were speech and language therapists (SLT) n=131; occupational therapists (OT) n=63; physiotherapists (PT) n=57; paediatricians n=50; dieticians n=40; nurses n=32 and health visitors n=14.

**Results:**

Most interventions were used commonly across diagnoses. However, some interventions were used more commonly with specific conditions, for example, positioning (CP 85%, DS 70%, ASD 23%, strategies/programmes aimed at changing behaviour at mealtimes (ASD 52%, CP 8%, DS 11%); visual supports (ASD 58%, CP 0%, DS 21%). HPs reported using a broad range of interventions, SLTs (mean=13.9), dieticians (12.3), OTs (12.7) and paediatricians (11.1). There was overlap between intervention use and HP role, for example, positioning (100% PT, 97% SLT, 94% OT, 73% paediatricians and 69% nurses).

**Conclusions:**

Interdisciplinary working is common when managing EDSD, with all HP types using multiple interventions. A child’s main diagnosis does not substantially influence intervention use, and the individual context of each child requires consideration in intervention selection. Study findings have supported development of the FEEDS Toolkit for use in feeding services.

**WHAT IS ALREADY KNOWN ON THIS TOPIC**

**WHAT THIS STUDY ADDS**

- Findings in this study describe current intervention practices within the feeding multidisciplinary team (MDT) and suggest that specific healthcare professions may recommend and use interventions outside their traditional professional roles, demonstrating interdisciplinary working and the versatility of feeding teams.
- Results support taking a child-centred approach to intervention use, considering not only a child’s main neurodevelopmental diagnoses but their whole context.

**HOW THIS STUDY MIGHT AFFECT RESEARCH, PRACTICE OR POLICY**

- Findings from this study supported the creation of the Focus on Early Eating, Drinking and Swallowing Toolkit, an informative shared decision aid tool used by parent carers and the MDT.
- The Toolkit is now being used in the UK NHS; training for professionals to use the Toolkit in their practice is now available through: feeds@ncl.ac.uk.

## Introduction

 Adequate nutrition is important for the development, growth and health of children.^1^ Children with neurodevelopmental conditions and children with neurodisability can often have eating, drinking or swallowing difficulties that impact their ability to meet their nutritional requirements, leading to impaired growth and poorer health outcomes.^2^,^4^

The FEEDS (Focus on Early Eating, Drinking and Swallowing) review aimed to find and appraise the evidence on interventions for eating, drinking and swallowing difficulties (EDSD) for children with neurodisability and their use, and to determine which interventions are currently recommended by National Health Service (NHS) professions; and which are used by parent carers.^5^ These aims were met by using multiple study methods including systematic reviews, focus groups with stakeholders, surveys and consultation workshops.^5^,^7^

The FEEDS national survey aimed to ‘establish current UK clinical practice in relation to the interventions recommended and used for EDSD in young children with neurodisability and the outcomes measured, from the perspective of parents and health and education professionals.^5^ Participants included (1) healthcare professionals (HPs) working with children with neurodisability and EDSD and (2) parent carers of children with neurodisability and EDSD. They were asked about their recommendation (professionals) or use (parents) of 25 interventions, perception of those interventions and the importance of specific outcomes. Previous analysis of responses determined intervention use by EDSD aetiology (physical, non-physical, and mixed physical and non-physical impairment) and intervention use by HPs.^5 6^ Subsequently, practitioners asked about intervention use by HP type and by condition. We conducted analyses in response to practitioner questions and to support development of the FEEDS Toolkit. The FEEDS Toolkit, which describes the interventions, how to use them and ways of measuring their impacts, was subsequently developed. The Toolkit provides a way to facilitate a shared decision-making approach for HPs and parent carers to determine which interventions to try to achieve specific agreed outcomes regarding feeding.

The findings from this study aimed to further analyse the national survey responses from the FEEDS review to inform on current intervention practices within the NHS and determine whether intervention use varied by children’s main diagnosis and whether intervention recommendations varied by HP role.^5 8^

## Methods

Parent carers of children up to the age of 12 with neurodisability and EDSD and HPs working with children and young people aged 0–18 years with neurodisability and EDSD were recruited to the FEEDS national survey via parent carer networks and charities and relevant national professional bodies. Multiple sources of recruitment were chosen to maximise number of participants and to reduce bias.

The survey was designed by the research team following best practice in survey design.^9^ Data were collected regarding participant demographic characteristics, experiences of supporting children with EDSD, interventions (usage, effectiveness, acceptability) and important outcomes. Most questions offered fixed-choice responses, with opportunities for free-text responses. Recruitment was from March to September 2018 and the questionnaire was sent via email (hosted on Qualtrics^10^), with paper versions also available. Please see online supplemental material 1 for the previously published questionnaire for HPs and parent carers.^5 6^ The process leading to the creation of the FEEDS national survey and full recruitment strategies are outlined elsewhere.^5 6^

Exemplar parent-reported main neurodevelopmental diagnoses representing varied aetiologies of EDSD, and with over 10 respondents, were selected for analysis: cerebral palsy (CP n=30), autism spectrum disorder (ASD n=183) and Down syndrome (DS n=69). Information about any additional neurodevelopmental diagnoses was not requested. HP roles were selected for analysis where data were available for more than 10 respondents: speech and language therapist (SLT n=131), occupational therapist (OT n=63), physiotherapist (PT n=57), paediatrician (n=50), dietician (n=40), nurse (n=32) and health visitor (HV n=14).

Fixed-choice responses regarding the participant’s use of the 25 interventions included (see online supplemental material 2) were analysed using SPSS V.29.0.1.0.^11^ Statistical analysis was not conducted on the data due to the small cohort sizes. Missing data were not included in analysis and percentages of valid data were used to compare responses across cohorts and interventions.

### Patient and public involvement

Stakeholder involvement in the development of the national survey has been previously reported.^5 7^ Parent carers and HPs participated in FEEDS review as coapplicants for funding, as study investigators and via advisory groups.^7^ Stakeholders continued to be involved at further stages of the FEEDS review (eg, national workshop and dissemination). Parents and professionals have also contributed to FEEDS Toolkit design.

## Results

### Participant characteristics

Data on included respondents’ characteristics can be seen in table 1. 86.8% of HP worked within the NHS, and 93.6% of parent-carers were mothers. Most (88.3%) respondents identified their ethnicity as ‘white’. All regions in England were represented; there were a small number of responses from other UK nations.

**Table 1:** Participant characteristics

Healthcare professionals	n (%)	Parents	n (%)
Employer		Role	
National Health Service Trust	336 (86.8)	Mother	264 (93.6)
Education	20 (5.2)	Father	12 (4.3)
Independent practitioner	15 (3.9)	Relative	1 (0.4)
Voluntary sector	11 (2.8)	Carer	4 (1.4)
Other	4 (1.0)	Other	1 (0.4)
Region of UK		Region of UK	
Midlands	53 (13.7)	Midlands	51 (18.1)
North East England	34 (8.8)	North East England	53 (18.8)
North West England	25 (6.5)	North West England	24 (8.5)
South East England including London	154 (39.8)	South East England including London	69 (24.5)
South West England	20 (5.2)	South West England	31 (11.0)
Yorkshire and Humber	57 (14.7)	Yorkshire and Humber	30 (10.6)
Northern Ireland	10 (2.6)	Northern Ireland	5 (1.8)
Scotland	19 (4.9)	Scotland	13 (4.6)
Wales	15 (3.9)	Wales	6 (2.1)
		Ethnicity	
		White	249 (88.3)
		Black/African/Caribbean/Black British	5 (1.8)
		Mixed/Multiple Ethnic Groups	7 (2.5)
		Prefer not to say	3 (1.1)

### Parent carer responses

Most parent carers reported using multiple interventions, with a similar mean number of interventions used in autistic children (6.3, SD=4.0), children with CP (mean=6.8, SD=4.3) and children with DS (7.0, SD=4.6).

Many interventions were used commonly irrespective of main neurodevelopmental diagnosis; for example, food and drink modification (ASD 50%, CP 67%, DS 83%), desensitising for food avoidance (ASD 55%, CP 38%, DS 61%) and modification of utensils (ASD 38%, CP 42%, DS 57%). However, some interventions were more commonly used with children with a specific condition: positioning (ASD 23%, CP 85%, DS 70%), strategies/programmes aimed at changing behaviour at mealtimes (ASD 52%, CP 8%, DS 11%) and visual supports (ASD 58%, CP 0%, DS 21%) (table 2).

**Table 2:** Intervention use by child’s main diagnosis

Intervention	Autism spectrum disorderN=183n (%)*	Cerebral palsyN=30n (%)*	Down syndromeN=69n (%)*
Counselling	12 (7)	1 (4)	1 (2)
Desensitisation programme for food avoidance	88 (55)	9 (38)	34 (61)
Desensitisation programme for oral sensations	24 (15)	9 (38)	17 (30)
Energy supplements	27 (16)	8 (31)	12 (21)
Enhancing child/feeder communication strategies at mealtimes	88 (53)	9 (38)	20 (35)
Food or drink modification	85 (50)	18 (67)	48 (83)
Hand over hand prompting	52 (32)	11 (46)	33 (58)
Sharing information on the impact of movement difficulties on eating and drinking	14 (9)	11 (46)	17 (30)
Sharing information on the impact of sensory difficulties on eating and drinking	92 (58)	6 (25)	19 (34)
Manoeuvres	7 (4)	4 (15)	17 (29)
Medication	24 (14)	21 (78)	31 (53)
Modelling	23 (15)	0 (0)	11 (20)
Modification of the environment	87 (51)	10 (38)	11 (19)
Modification of utensils	65 (38)	11 (42)	33 (57)
Modifying social eating and drinking opportunities	45 (28)	5 (21)	14 (25)
Oral-motor exercises	28 (16)	6 (22)	30 (52)
Pacing of food at mealtimes	30 (18)	8 (32)	17 (30)
Positioning	41 (23)	23 (85)	43 (70)
Schedule of meals	72 (42)	5 (19)	12 (21)
Sensorimotor therapies	4 (3)	3 (13)	4 (7)
Sensory aids	22 (13)	3 (12)	14 (24)
Sensory stimulation	20 (13)	11 (46)	16 (29)
Strategies/programmes aimed at changing behaviour at mealtimes	82 (52)	2 (8)	6 (11)
Training to wait for a child’s cues for feeding	21 (13)	9 (35)	9 (16)
Visual supports	91 (58)	0 (0)	12 (21)

* Totals and percentages vary due to missing data for some interventions. Missing data: ASD (mean=10%, SD=3%), CP (mean=16%, SD=4%), DS (mean=17%, SD=2%).

ASDautism spectrum disorderCPcerebral palsy

### HP responses

HPs used or recommended multiple interventions. SLTs, paediatricians and OTs used the highest number of interventions: SLT mean=13.9 interventions (SD=4.0), OT 12.7 (SD=3.6), dieticians 12.3 (SD=5.3), paediatricians 11.1 (SD=6.0), and HVs, nurses and PTs recommended fewer interventions (9.4 (SD=6.9), 7.5 (SD=4.0), 5.9 (SD=4.9) respectively).

Some interventions were commonly recommended by multiple HP roles, for example, positioning was recommended by 97% of SLTs, 94% of OTs, 100% PTs, 73% of paediatricians and 69% of nurses. However, some interventions were more commonly recommended in the context of specific HP roles, for example, energy supplements (100% of dieticians, 78% of paediatricians) and waiting for cues (75% of SLT, 82% of HV). A small number of interventions were rarely recommended by any HP role, for example, sensorimotor therapy (7% of SLT, 6% of OT, 1% of HV and 0% of dieticians, PT, paediatricians and nurses) (table 3).

**Table 3:** Intervention use by healthcare professional role

Intervention	SLTN=183n (%)*	DieticiansN=30n (%)*	OTN=63n (%)*	PTN=57n (%)*	PaediatriciansN=50n (%)*	NursesN=32n (%)*	HVN=14n (%)*
Counselling	33 (33)	17 (63)	18 (35)	6 (15)	23 (66)	9 (39)	5 (50)
Desensitisation programme for food avoidance	79 (79)	21 (78)	45 (87)	10 (25)	19 (54)	9 (39)	6 (55)
Desensitisation programme for oral sensations	52 (52)	9 (33)	27 (52)	5 (13)	9 (26)	3 (13)	1 (9)
Energy supplements	14 (14)	28 (100)	5 (9)	2 (5)	28 (78)	4 (16)	1 (9)
Enhancing child/feeder communication strategies at mealtimes	80 (79)	15 (56)	29 (54)	6 (15)	7 (20)	7 (29)	6 (55)
Food or drink modification	102 (98)	28 (97)	33 (58)	12 (28)	27 (75)	15 (56)	5 (50)
Hand over hand prompting	40 (40)	5 (19)	43 (83)	14 (35)	11 (31)	6 (26)	3 (27)
Sharing information on the impact of movement difficulties on eating and drinking	76 (76)	12 (44)	41 (80)	37 (95)	26 (74)	7 (30)	3 (30)
Sharing information on the impact of sensory difficulties on eating and drinking	80 (81)	22 (82)	44 (86)	13 (33)	24 (69)	10 (44)	5 (50)
Manoeuvres	90 (79)	8 (24)	22 (37)	12 (27)	15 (38)	2 (7)	0 (0)
Medication	52 (48)	17 (53)	10 (18)	12 (27)	35 (92)	27 (90)	2 (18)
Modelling	21 (21)	9 (36)	13 (26)	2 (5)	3 (9)	1 (4)	0 (0)
Modification of the environment	89 (87)	20 (71)	48 (84)	18 (42)	16 (44)	8 (31)	5 (50)
Modification of utensils	78 (76)	8 (28)	54 (95)	15 (35)	18 (50)	8 (31)	3 (27)
Modifying social eating and drinking opportunities	30 (30)	12 (44)	26 (49)	0 (0)	15 (43)	5 (21)	4 (36)
Oral-motor exercises	44 (40)	5 (16)	27 (47)	6 (14)	12 (32)	2 (7)	3 (21)
Pacing of food at mealtimes	99 (97)	16 (57)	26 (48)	5 (12)	16 (46)	3 (13)	7 (70)
Positioning	121 (97)	11 (32)	59 (94)	55 (100)	38 (73)	22 (69)	6 (46)
Schedule of meals	54 (51)	29 (97)	18 (32)	4 (9)	14 (38)	15 (52)	3 (30)
Sensorimotor therapies	7 (7)	0 (0)	3 (6)	0 (0)	0 (0)	0 (0)	1 (9)
Sensory aids	15 (15)	3 (11)	7 (13)	4 (9)	16 (44)	9 (35)	1 (9)
Sensory stimulation	31 (31)	2 (7)	12 (23)	5 (13)	1 (3)	6 (26)	1 (9)
Strategies/programmes aimed at changing behaviour at mealtimes	38 (38)	25 (93)	29 (57)	3 (8)	17 (49)	10 (44)	9 (82)
Training to wait for a child’s cues for feeding	76 (75)	11 (39)	11 (20)	9 (22)	9 (26)	6 (25)	9 (82)
Visual supports	54 (55)	8 (32)	39 (77)	3 (8)	11 (31)	3 (13)	3 (27)

Missing data: SLT (mean=21%, SD=5%), dieticians (mean=29%, SD=6%), OT (mean=14%, SD=5%), PT (mean=27%, SD=6%), paediatrician (mean=30%, SD=6%), nurse (mean=21%, SD=9%) and HV (mean=23%, SD=5%).

* Totals and percentages vary due to missing data for some of the interventions.

HVhealth visitorOToccupational therapistPTphysiotherapistSLTspeech and language therapist

## Discussion

This study aimed to investigate intervention use by parent carers of children with specific neurodevelopmental diagnoses and by different HPs in their roles within the feeding multidisciplinary team (MDT). The results provide information about current UK feeding services and their intervention use. The results have also been used to develop the FEEDS Toolkit, ensuring it is relevant to a wide range of HPs, and to parents of children with a range of diagnoses.

Responses show many interventions for children’s EDSD were used commonly across children with the different neurodevelopmental diagnoses. In contrast, some interventions were found to be more commonly used with children with specific conditions, for example, positioning in children with CP and DS, and strategies/programmes aimed at changing behaviour at mealtimes in autistic children. In comparison to previously reported findings regarding children with physical and mixed EDSD, and non-physical EDSD, a substantial difference in intervention use cannot be seen, for example, positioning reported as used by 61.8% of parent carers of children with physical and mixed EDSD, in comparison to 22.4% of parent carers of children with non-physical EDSD.^6^ This suggests that a child’s main neurodevelopmental diagnosis is not the main contributing factor determining intervention use, and the aetiology of the child’s EDSD, in addition to individual medical and social contexts, should inform intervention selection.

Our findings regarding intervention use by specific UK NHS HP role for children with EDSD may be related to professional availability to families and MDT working. The findings suggest that the Toolkit may be useful to MDT HPs, as while HPs most commonly recommend interventions related to their specialty, there was substantial evidence of HPs recommending interventions outside the scope of their traditionally perceived role. The versatility of the interventions included in the FEEDS Toolkit will support the future uptake in the NHS, despite regional variation in HP access and will provide a framework and opportunity for HPs to use the broad range of included interventions with parents. Some interventions were rarely recommended/used by any HP role. This could reflect HPs’ views on current evidence for intervention effectiveness or the lack of training/support to implement their use—the FEEDS Toolkit content may enable professionals to speak with MDT colleagues about interventions they are less familiar with and discuss a broader range of interventions with parents.

Limitations of this study include the small and unequal cohort sizes, both for main diagnoses groups and HP roles, precluding statistical comparisons. Additionally, the presence of comorbidities, coexisting diagnoses and their influence on feeding could not be considered, as only information about main neurodevelopmental diagnosis was collected. These limitations may have resulted in low reported use of some interventions (eg, no reported use of visual supports for children with CP). In the future, data collected through studies of FEEDS Toolkit use will be used to understand which interventions are used by parents of children with a range of diagnoses. Finally, despite using multiple methods to distribute the survey,^5^ respondents were mostly mothers, and most were white. Representation across the four nations was limited. Future data collected via the FEEDS Toolkit directly will allow for the collection of more representative data.

Findings from this study have contributed to the FEEDS Toolkit design by showing the range of interventions used in clinical practice. The lack of observable patterns relating to child main neurodevelopmental diagnosis further emphasises the importance of an individualised and child-centred approach to intervention use, rather than focussing on a child’s main diagnosis. Intervention use by specific HPs will inform how the FEEDS Toolkit might be implemented within already existing feeding services, and which HPs should be involved in using the FEEDS Toolkit and its further development and testing. Future data collected through evaluation of FEEDS Toolkit use may give information about the range of interventions used by parents of children and young people with specific conditions or a number of conditions.

## Conclusion

HPs within feeding teams work in an interdisciplinary manner and there is often overlap across HP roles and intervention recommendation/use. A child’s main neurodevelopmental diagnosis does not substantially influence intervention use and should not be the main factor of consideration when deciding interventions to use—an individualised approach should be adopted. These findings can be used to plan clinical provision, and how UK and international health professionals work with young people and families. The findings influenced FEEDS Toolkit design and should improve this resource that supports parent carers and HPs to work collaboratively and make decisions about which interventions to use to improve feeding.

## supplementary material

10.1136/bmjpo-2023-002394 online supplemental file 1

10.1136/bmjpo-2023-002394 online supplemental file 2

## Data Availability

Data are available on reasonable request.
