# Supplementary material for: Informing creation of the FEEDS Toolkit to support parent-delivered interventions for eating, drinking and swallowing difficulties in young children with neurodisability: intervention use by neurodevelopmental diagnosis and healthcare professional role
Source: BMJ Paediatr Open. 2024 Aug 24;8(1):e002394. doi: 10.1136/bmjpo-2023-002394 (PMC11733779; doi:10.1136/bmjpo-2023-002394)
Supplement: online supplemental file 1 [file bmjpo-8-1-s001.pdf]

Supplementary Material 1- National Survey Questionnaire

This survey has previously been published as supplementary material (1,2):

1. Parr J, Pennington L, Taylor H, Craig D, Morris C, McConachie H, et al. Parent-delivered interventions used at home to improve eating, drinking and swallowing in children with neurodisability: The feeds mixed-methods study. Health Technol Assess (Rockv). 2021;25(22):1–208.
2. Taylor H, Pennington L, Craig D, Morris C, Mcconachie H, Cadwgan J, et al. Children with neurodisability and feeding difficulties: a UK survey of parent-delivered interventions. 2021;5:1095.

National survey questionnaire

The health professional's version of the national survey is shown below.
The parent version differed from the health professional's version in the following ways. It asked for additional information about the child being reported on: age; education setting; diagnosis; age EDSD started; whether there were continued EDSD; when EDSD resolved; cause of EDSD; involvement of health professionals; whether explanation / diagnosis given in relation to EDSD. It also asked the following questions in relation to each intervention: whether they had used it; what age their child was when they started using it; how long they used it for; which professionals recommended it; whether they thought it worked; whether they received training; and whether it was acceptable to deliver at home.

The education professional's version differed from the health professional's version in the following way. It asked whether respondents had been involved in delivering strategies aimed at improving EDSD. It also asked the following questions in relation to each intervention: whether they had been involved in using the strategy; whether they received training; whether they thought it worked; how long, on average, they thought it took to see changes; and whether it was acceptable to deliver in school.

FEEDS Health Professionals Survey

Please tell us some information about you:

1. What is your role?	Speech and Language Therapist ☐<br>Dietitian ☐<br>Occupational Therapist ☐<br>Physiotherapist ☐<br>Clinical Psychologist ☐<br>Paediatrician ☐<br>Gastroenterologist ☐<br>Nurse ☐<br>Health Visitor ☐<br>Other (please specify)________________________________
2. How long have you been working with young people with neurodevelopmental disorders / neurodisability who experience eating, drinking or swallowing difficulties?	_______ years
3. In which region of the UK do you work?	North East England ☐<br>North West England ☐<br>South East England including London ☐<br>South West England ☐<br>Midlands ☐<br>Scotland ☐<br>Northern Ireland ☐<br>Wales ☐
4. Who is your employer?	NHS Trust ☐<br>If so, which NHS Trust? (please specify):________________<br>____<br>Education ☐<br>Voluntary Sector ☐<br>Independent Practitioner ☐<br>Other (please specify):________________________________

Please tell us some information about your role with children who have eating, drinking and swallowing difficulties:

5. What age of children do you work with?<br><br>(Please tick all that apply)	0-6 months ☐<br>7-11 months ☐<br><br>1-3 years ☐<br><br>4-8 years ☐<br><br>9 years and above ☐
6. What type of eating, drinking and swallowing difficulties do you work with?	Physical difficulties ☐<br>*(Reduced control of the muscles of the lips, tongue, mouth and throat)*<br><br>Non-physical difficulties ☐<br>*(Sensory sensitivity leading to restricted or selective eating and rituals associated with food or mealtimes)*<br><br>Both physical and non-physical difficulties ☐
7. Which setting(s) do you work in?<br><br>(Please tick all that apply)	Community Services ☐<br><br>Secondary Care ☐<br><br>Tertiary Care ☐<br><br>Education ☐<br><br>Other (please specify) ____________________________
8. Which other professionals are involved with children's eating, drinking and swallowing difficulties locally?	Speech and Language Therapist ☐<br><br>Dietitian ☐<br><br>Occupational Therapist ☐<br><br>Physiotherapist ☐<br><br>Clinical Psychologist ☐<br><br>Paediatrician ☐<br><br>Gastroenterologist ☐<br><br>Nurse ☐<br><br>Health Visitor ☐<br><br>Education ☐<br><br>Other (please specify):____________________________

9. Do you work with parents to deliver interventions?	Yes ☐<br>No ☐
9a. IF NO, who supports parents to deliver interventions locally?<br><br>(Please tick all that apply)	Speech and Language Therapist ☐<br>Dietitian ☐<br>Occupational Therapist ☐<br>Physiotherapist ☐<br>Clinical Psychologist ☐<br>Paediatrician ☐<br>Gastroenterologist ☐<br>Nurse ☐<br>Health Visitor ☐<br>Education ☐<br>Other (please specify):____________________________<br>Don't know ☐
10. Do you support education staff to deliver interventions?	Yes ☐<br>No ☐
10a. IF NO, who supports education staff to deliver interventions locally?<br><br>(Please tick all that apply)	Speech and Language Therapist ☐<br>Dietitian ☐<br>Occupational Therapist ☐<br>Physiotherapist ☐<br>Clinical Psychologist ☐<br>Paediatrician ☐<br>Gastroenterologist ☐<br>Nurse ☐<br>Health Visitor ☐<br>Other (please specify):____________________________<br>Don't know ☐

In the next section we would like to know about the interventions you use with children with eating, drinking and swallowing difficulties. We appreciate that many interventions are used together as part of an overall treatment plan, but we are interested in knowing whether you use each of the component interventions listed (whether this is on its own or as part of a treatment plan). Some interventions are broad and some more specific. This study is about interventions parents can use at home and so we are not covering tube feeding (nasogastric or gastrostomy).

For each of the strategies listed in the tables following, please first answer whether you use or used it.

- If you answer 'yes' for that strategy, please go onto answer all the questions listed in the columns across the page in relation to that strategy.
- If you answer 'no' for that strategy, please go onto the next question below relating to the next strategy.

Intervention for eating, drinking or swallowing difficulties	Do you use it?	What age range(s) do you use this with? (tick all that apply)	Do you provide ongoing support with this intervention? (tick one)	Which clinical group(s) do you use this with? (tick one)	Where is it delivered? (tick all that apply)	In your opinion, is it effective? (tick one)	Over what timescale does change usually occur? (tick one)	Is training given to parents and school / nursery? (tick Y/N for each)
**11. Any type of positioning e.g. trunk stability, head movement, specialist seating**	Yes ☐<br>No ☐<br>**If NO**, please move to question 12 below	0-6 months ☐<br>7-11 months ☐<br>1-3 years ☐<br>4-8 years ☐<br>9 years and above ☐	One-off intervention ☐<br>Time limited programme ☐<br>Ongoing support ☐	Physical difficulties ☐<br>Non-physical difficulties ☐<br>Both ☐	Home ☐<br>Nursery / playgroup ☐<br>School ☐<br>Hospital ☐<br>Other: ___________	Yes ☐<br>No ☐<br>Sometimes ☐<br>(please state when it is effective): ___________ ___________ ___________	0-3 months ☐<br>4-6 months ☐<br>7-9 months ☐<br>10-12 months ☐<br>Over 1 year ☐	Parents: Yes ☐ No ☐<br>School / Nursery: Yes ☐ No ☐
**12. Any type of manoeuvre e.g. jaw support, chin tuck**	Yes ☐<br>No ☐<br>**If NO**, please move to question 13 below	0-6 months ☐<br>7-11 months ☐<br>1-3 years ☐<br>4-8 years ☐<br>9 years and above ☐	One-off intervention ☐<br>Time limited programme ☐<br>Ongoing support ☐	Physical difficulties ☐<br>Non-physical difficulties ☐<br>Both ☐	Home ☐<br>Nursery / playgroup ☐<br>School ☐<br>Hospital ☐<br>Other: ___________	Yes ☐<br>No ☐<br>Sometimes ☐<br>(please state when it is effective): ___________ ___________ ___________	0-3 months ☐<br>4-6 months ☐<br>7-9 months ☐<br>10-12 months ☐<br>Over 1 year ☐	Parents: Yes ☐ No ☐<br>School / Nursery: Yes ☐ No ☐

Intervention for eating, drinking or swallowing difficulties	Do you use it?	What age range(s) do you use this with? (tick all that apply)	Do you provide ongoing support with this intervention? (tick one)	Which clinical group(s) do you use this with? (tick one)	Where is it delivered? (tick all that apply)	In your opinion, is it effective? (tick one)	Over what timescale does change usually occur? (tick one)	Is training given to parents and school / nursery? (tick Y/N for each)
**13. Any type of oral motor exercises to improve the child's physical ability e.g. improving muscle tone, tongue strengthening**	Yes ☐<br>No ☐<br>**If NO**, please move to question 14 below	0-6 months ☐<br>7-11 months ☐<br>1-3 years ☐<br>4-8 years ☐<br>9 years and above ☐	One-off intervention ☐<br>Time limited programme ☐<br>Ongoing support ☐	Physical difficulties ☐<br>Non-physical difficulties ☐<br>Both ☐	Home ☐<br>Nursery / playgroup ☐<br>School ☐<br>Hospital ☐<br>Other:<br>____________	Yes ☐<br>No ☐<br>Sometimes ☐<br>(please state when it is effective):<br>____________<br>____________<br>____________	0-3 months ☐<br>4-6 months ☐<br>7-9 months ☐<br>10-12 months ☐<br>Over 1 year ☐	Parents:<br>Yes ☐<br>No ☐<br>School / Nursery:<br>Yes ☐<br>No ☐
**14. Any type of medication e.g. for epilepsy, pain, drooling, tone, gastro-oesophageal reflux**	Yes ☐<br>No ☐<br>**If NO**, please move to question 15 below	0-6 months ☐<br>7-11 months ☐<br>1-3 years ☐<br>4-8 years ☐<br>9 years and above ☐	One-off intervention ☐<br>Time limited programme ☐<br>Ongoing support ☐	Physical difficulties ☐<br>Non-physical difficulties ☐<br>Both ☐	Home ☐<br>Nursery / playgroup ☐<br>School ☐<br>Hospital ☐<br>Other:<br>____________	Yes ☐<br>No ☐<br>Sometimes ☐<br>(please state when it is effective):<br>____________<br>____________<br>____________	0-3 months ☐<br>4-6 months ☐<br>7-9 months ☐<br>10-12 months ☐<br>Over 1 year ☐	Parents:<br>Yes ☐<br>No ☐<br>School / Nursery:<br>Yes ☐<br>No ☐

Intervention for eating, drinking or swallowing difficulties	Do you use it?	What age range(s) do you use this with? (tick all that apply)	Do you provide ongoing support with this intervention? (tick one)	Which clinical group(s) do you use this with? (tick one)	Where is it delivered? (tick all that apply)	In your opinion, is it effective? (tick one)	Over what timescale does change usually occur? (tick one)	Is training given to parents or school / nursery? (tick Y/N for each)
**15. Schedule of meals to promote appetite or increase predictability e.g. written meal plan**	Yes ☐<br>No ☐<br>**If NO**, please move to question 16 below	0-6 months ☐<br>7-11 months ☐<br>1-3 years ☐<br>4-8 years ☐<br>9 years and above ☐	One-off intervention ☐<br>Time limited programme ☐<br>Ongoing support ☐	Physical difficulties ☐<br>Non-physical difficulties ☐<br>Both ☐	Home ☐<br>Nursery / playgroup ☐<br>School ☐<br>Hospital ☐<br>Other: ____________	Yes ☐<br>No ☐<br>Sometimes ☐<br>(please state when it is effective): ____________ ____________ ____________	0-3 months ☐<br>4-6 months ☐<br>7-9 months ☐<br>10-12 months ☐<br>Over 1 year ☐	Parents: Yes ☐ No ☐<br>School / Nursery: Yes ☐ No ☐
**16. Any type of food or drink modification e.g. consistency, temperature, taste, volume**	Yes ☐<br>No ☐<br>**If NO**, please move to question 17 below	0-6 months ☐<br>7-11 months ☐<br>1-3 years ☐<br>4-8 years ☐<br>9 years and above ☐	One-off intervention ☐<br>Time limited programme ☐<br>Ongoing support ☐	Physical difficulties ☐<br>Non-physical difficulties ☐<br>Both ☐	Home ☐<br>Nursery / playgroup ☐<br>School ☐<br>Hospital ☐<br>Other: ____________	Yes ☐<br>No ☐<br>Sometimes ☐<br>(please state when it is effective): ____________ ____________ ____________	0-3 months ☐<br>4-6 months ☐<br>7-9 months ☐<br>10-12 months ☐<br>Over 1 year ☐	Parents: Yes ☐ No ☐<br>School / Nursery: Yes ☐ No ☐

Intervention for eating, drinking or swallowing difficulties	Do you use it?	What age range(s) do you use this with? (tick all that apply)	Do you provide ongoing support with this intervention? (tick one)	Which clinical group(s) do you use this with? (tick one)	Where is it delivered? (tick all that apply)	In your opinion, is it effective? (tick one)	Over what timescale does change usually occur? (tick one)	Is training given to parents or school / nursery? (tick Y/N for each)
**17.Any type of modification to eating and drinking utensils**	Yes ☐<br>No ☐<br>**If NO**, please move to question 18 below	0-6 months ☐<br>7-11 months ☐<br>1-3 years ☐<br>4-8 years ☐<br>9 years and above ☐	One-off intervention ☐<br>Time limited programme ☐<br>Ongoing support ☐	Physical difficulties ☐<br>Non-physical difficulties ☐<br>Both ☐	Home ☐<br>Nursery / playgroup ☐<br>School ☐<br>Hospital ☐<br>Other: ____________	Yes ☐<br>No ☐<br>Sometimes ☐<br>(please state when it is effective): ____________ ____________ ____________	0-3 months ☐<br>4-6 months ☐<br>7-9 months ☐<br>10-12 months ☐<br>Over 1 year ☐	Parents: Yes ☐ No ☐<br>School / Nursery: Yes ☐ No ☐
**18. Any type of modification to the environment at mealtimes e.g. noise, lighting, reduced social demands**	Yes ☐<br>No ☐<br>**If NO**, please move to question 19 below	0-6 months ☐<br>7-11 months ☐<br>1-3 years ☐<br>4-8 years ☐<br>9 years and above ☐	One-off intervention ☐<br>Time limited programme ☐<br>Ongoing support ☐	Physical difficulties ☐<br>Non-physical difficulties ☐<br>Both ☐	Home ☐<br>Nursery / playgroup ☐<br>School ☐<br>Hospital ☐<br>Other: ____________	Yes ☐<br>No ☐<br>Sometimes ☐<br>(please state when it is effective): ____________ ____________ ____________	0-3 months ☐<br>4-6 months ☐<br>7-9 months ☐<br>10-12 months ☐<br>Over 1 year ☐	Parents: Yes ☐ No ☐<br>School / Nursery: Yes ☐ No ☐

Intervention for eating, drinking or swallowing difficulties	Do you use it?	What age range(s) do you use this with? (tick all that apply)	Do you provide ongoing support with this intervention? (tick one)	Which clinical group(s) do you use this with? (tick one)	Where is it delivered? (tick all that apply)	In your opinion, is it effective? (tick one)	Over what timescale does change usually occur? (tick one)	Is training given to parents or school / nursery? (tick Y/N for each)
**19. Any type of sensory aid e.g. glasses, hearing aids**	Yes ☐<br>No ☐<br>**If NO**, please move to question 20 below	0-6 months ☐<br>7-11 months ☐<br>1-3 years ☐<br>4-8 years ☐<br>9 years and above ☐	One-off intervention ☐<br>Time limited programme ☐<br>Ongoing support ☐	Physical difficulties ☐<br>Non-physical difficulties ☐<br>Both ☐	Home ☐<br>Nursery / playgroup ☐<br>School ☐<br>Hospital ☐<br>Other: ____________	Yes ☐<br>No ☐<br>Sometimes ☐<br>(please state when it is effective):<br>____________<br>____________<br>____________	0-3 months ☐<br>4-6 months ☐<br>7-9 months ☐<br>10-12 months ☐<br>Over 1 year ☐	Parents: Yes ☐<br>No ☐<br>School / Nursery: Yes ☐<br>No ☐
**20. Use of energy supplements**	Yes ☐<br>No ☐<br>**If NO**, please move to question 21 below	0-6 months ☐<br>7-11 months ☐<br>1-3 years ☐<br>4-8 years ☐<br>9 years and above ☐	One-off intervention ☐<br>Time limited programme ☐<br>Ongoing support ☐	Physical difficulties ☐<br>Non-physical difficulties ☐<br>Both ☐	Home ☐<br>Nursery / playgroup ☐<br>School ☐<br>Hospital ☐<br>Other: ____________	Yes ☐<br>No ☐<br>Sometimes ☐<br>(please state when it is effective):<br>____________<br>____________<br>____________	0-3 months ☐<br>4-6 months ☐<br>7-9 months ☐<br>10-12 months ☐<br>Over 1 year ☐	Parents: Yes ☐<br>No ☐<br>School / Nursery: Yes ☐<br>No ☐

Intervention for eating, drinking or swallowing difficulties	Do you use it?	What age range(s) do you use this with? (tick all that apply)	Do you provide ongoing support with this intervention? (tick one)	Which clinical group(s) do you use this with? (tick one)	Where is it delivered? (tick all that apply)	In your opinion, is it effective? (tick one)	Over what timescale does change usually occur? (tick one)	Is training given to parents or school / nursery? (tick Y/N for each)
**21. Training to wait for child cues for feeding**	Yes ☐<br>No ☐<br>**If NO**, please move to question 22 below	0-6 months ☐<br>7-11 months ☐<br>1-3 years ☐<br>4-8 years ☐<br>9 years and above ☐	One-off intervention ☐<br>Time limited programme ☐<br>Ongoing support ☐	Physical difficulties ☐<br>Non-physical difficulties ☐<br>Both ☐	Home ☐<br>Nursery / playgroup ☐<br>School ☐<br>Hospital ☐<br>Other: ____________	Yes ☐<br>No ☐<br>Sometimes ☐<br>(please state when it is effective): ____________<br>____________<br>____________	0-3 months ☐<br>4-6 months ☐<br>7-9 months ☐<br>10-12 months ☐<br>Over 1 year ☐	Parents: Yes ☐<br>No ☐<br>School / Nursery: Yes ☐<br>No ☐
**22. Pacing of food at mealtimes**	Yes ☐<br>No ☐<br>**If NO**, please move to question 23 below	0-6 months ☐<br>7-11 months ☐<br>1-3 years ☐<br>4-8 years ☐<br>9 years and above ☐	One-off intervention ☐<br>Time limited programme ☐<br>Ongoing support ☐	Physical difficulties ☐<br>Non-physical difficulties ☐<br>Both ☐	Home ☐<br>Nursery / playgroup ☐<br>School ☐<br>Hospital ☐<br>Other: ____________	Yes ☐<br>No ☐<br>Sometimes ☐<br>(please state when it is effective): ____________<br>____________<br>____________	0-3 months ☐<br>4-6 months ☐<br>7-9 months ☐<br>10-12 months ☐<br>Over 1 year ☐	Parents: Yes ☐<br>No ☐<br>School / Nursery: Yes ☐<br>No ☐

Intervention for eating, drinking or swallowing difficulties	Do you use it?	What age range(s) do you use this with? (tick all that apply)	Do you provide ongoing support with this intervention? (tick one)	Which clinical group(s) do you use this with? (tick one)	Where is it delivered? (tick all that apply)	In your opinion, is it effective? (tick one)	Over what timescale does change usually occur? (tick one)	Is training given to parents or school / nursery? (tick Y/N for each)
**23. Enhancing child / feeder communication strategies at mealtimes e.g. increasing choices**	Yes ☐<br>No ☐<br>**If NO**, please move to question 24 below	0-6 months ☐<br>7-11 months ☐<br>1-3 years ☐<br>4-8 years ☐<br>9 years and above ☐	One-off intervention ☐<br>Time limited programme ☐<br>Ongoing support ☐	Physical difficulties ☐<br>Non-physical difficulties ☐<br>Both ☐	Home ☐<br>Nursery / playgroup ☐<br>School ☐<br>Hospital ☐<br>Other: ____________	Yes ☐<br>No ☐<br>Sometimes ☐<br>(please state when it is effective): ____________<br>____________<br>____________	0-3 months ☐<br>4-6 months ☐<br>7-9 months ☐<br>10-12 months ☐<br>Over 1 year ☐	Parents: Yes ☐<br>No ☐<br>School / Nursery: Yes ☐<br>No ☐
**24. Modifying social eating or drinking opportunities**	Yes ☐<br>No ☐<br>**If NO**, please move to question 25 below	0-6 months ☐<br>7-11 months ☐<br>1-3 years ☐<br>4-8 years ☐<br>9 years and above ☐	One-off intervention ☐<br>Time limited programme ☐<br>Ongoing support ☐	Physical difficulties ☐<br>Non-physical difficulties ☐<br>Both ☐	Home ☐<br>Nursery / playgroup ☐<br>School ☐<br>Hospital ☐<br>Other: ____________	Yes ☐<br>No ☐<br>Sometimes ☐<br>(please state when it is effective): ____________<br>____________<br>____________	0-3 months ☐<br>4-6 months ☐<br>7-9 months ☐<br>10-12 months ☐<br>Over 1 year ☐	Parents: Yes ☐<br>No ☐<br>School / Nursery: Yes ☐<br>No ☐

Intervention for eating, drinking or swallowing difficulties	Do you use it?	What age range(s) do you use this with? (tick all that apply)	Do you provide ongoing support with this intervention? (tick one)	Which clinical group(s) do you use this with? (tick one)	Where is it delivered? (tick all that apply)	In your opinion, is it effective? (tick one)	Over what timescale does change usually occur? (tick one)	Is training given to parents or school / nursery? (tick Y/N for each)
**25. Hand over hand prompting i.e. leading the child by the hand to teach skills such as self-feeding**	Yes ☐<br>No ☐<br>**If NO**, please move to question 26 below	0-6 months ☐<br>7-11 months ☐<br>1-3 years ☐<br>4-8 years ☐<br>9 years and above ☐	One-off intervention ☐<br>Time limited programme ☐<br>Ongoing support ☐	Physical difficulties ☐<br>Non-physical difficulties ☐<br>Both ☐	Home ☐<br>Nursery / playgroup ☐<br>School ☐<br>Hospital ☐<br>Other: ____________	Yes ☐<br>No ☐<br>Sometimes ☐<br>(please state when it is effective):<br>______________<br>______________<br>______________	0-3 months ☐<br>4-6 months ☐<br>7-9 months ☐<br>10-12 months ☐<br>Over 1 year ☐	Parents: Yes ☐<br>No ☐<br>School / Nursery: Yes ☐<br>No ☐
**26. Counselling to parents / caregivers / staff of children with eating and drinking difficulties**	Yes ☐<br>No ☐<br>**If NO**, please move to question 27 below	0-6 months ☐<br>7-11 months ☐<br>1-3 years ☐<br>4-8 years ☐<br>9 years and above ☐	One-off intervention ☐<br>Time limited programme ☐<br>Ongoing support ☐	Physical difficulties ☐<br>Non-physical difficulties ☐<br>Both ☐	Home ☐<br>Nursery / playgroup ☐<br>School ☐<br>Hospital ☐<br>Other: ____________	Yes ☐<br>No ☐<br>Sometimes ☐<br>(please state when it is effective):<br>______________<br>______________<br>______________	0-3 months ☐<br>4-6 months ☐<br>7-9 months ☐<br>10-12 months ☐<br>Over 1 year ☐	Parents: Yes ☐<br>No ☐<br>School / Nursery: Yes ☐<br>No ☐

Intervention for eating, drinking or swallowing difficulties	Do you use it?	What age range(s) do you use this with? (tick all that apply)	Do you provide ongoing support with this intervention? (tick one)	Which clinical group(s) do you use this with? (tick one)	Where is it delivered? (tick all that apply)	In your opinion, is it effective? (tick one)	Over what timescale does change usually occur? (tick one)	Is training given to parents or school / nursery? (tick Y/N for each)
**27. Any type of desensitisation programme for food avoidance aimed at gradual acceptance of food e.g. messy play, sensory exploration, regular exposure to new foods**	Yes ☐<br>No ☐<br>**If NO**, please move to question 28 below	0-6 months ☐<br>7-11 months ☐<br>1-3 years ☐<br>4-8 years ☐<br>9 years and above ☐	One-off intervention ☐<br>Time limited programme ☐<br>Ongoing support ☐	Physical difficulties ☐<br>Non-physical difficulties ☐<br>Both ☐	Home ☐<br>Nursery / playgroup ☐<br>School ☐<br>Hospital ☐<br>Other: ____________	Yes ☐<br>No ☐<br>Sometimes ☐ (please state when it is effective): ____________ ____________ ____________	0-3 months ☐<br>4-6 months ☐<br>7-9 months ☐<br>10-12 months ☐<br>Over 1 year ☐	Parents: Yes ☐ No ☐<br>School / Nursery: Yes ☐ No ☐
**28. Any type of graded desensitisation activity aimed at reducing a child's excessive responses to**	Yes ☐<br>No ☐<br>**If NO**, please move to question 29 below	0-6 months ☐<br>7-11 months ☐<br>1-3 years ☐<br>4-8 years ☐<br>9 years and above ☐	One-off intervention ☐<br>Time limited programme ☐<br>Ongoing support ☐	Physical difficulties ☐<br>Non-physical difficulties ☐<br>Both ☐	Home ☐<br>Nursery / playgroup ☐<br>School ☐<br>Hospital ☐<br>Other: ____________	Yes ☐<br>No ☐<br>Sometimes ☐ (please state when it is effective): ____________ ____________	0-3 months ☐<br>4-6 months ☐<br>7-9 months ☐<br>10-12 months ☐<br>Over 1 year ☐	Parents: Yes ☐ No ☐<br>School / Nursery: Yes ☐ No ☐

**oral sensations linked to eating and drinking e.g. face massage, chewing non foods**						______________		
**Intervention for eating, drinking or swallowing difficulties**	**Do you use it?**	**What age range(s) do you use this with?<br>(tick all that apply)**	**Do you provide ongoing support with this intervention?<br>(tick one)**	**Which clinical group(s) do you use this with?<br>(tick one)**	**Where is it delivered?<br>(tick all that apply)**	**In your opinion, is it effective?<br>(tick one)**	**Over what timescale does change usually occur?<br>(tick one)**	**Is training given to parents or school / nursery?<br>(tick Y/N for each)**
**29. Any type of sensory stimulation e.g. tapping around mouth**	Yes ☐<br>No ☐<br>**If NO**, please move to question 30 below	0-6 months ☐<br>7-11 months ☐<br>1-3 years ☐<br>4-8 years ☐<br>9 years and above ☐	One-off intervention ☐<br>Time limited programme ☐<br>Ongoing support ☐	Physical difficulties ☐<br>Non-physical difficulties ☐<br>Both ☐	Home ☐<br>Nursery / playgroup ☐<br>School ☐<br>Hospital ☐<br>Other:<br>______________	Yes ☐<br>No ☐<br>Sometimes ☐<br>(please state when it is effective):<br>______________<br>______________<br>______________	0-3 months ☐<br>4-6 months ☐<br>7-9 months ☐<br>10-12 months ☐<br>Over 1 year ☐	Parents: Yes ☐<br>No ☐<br>School / Nursery: Yes ☐<br>No ☐
**30. Any type of sensori-motor therapy e.g. Facial Oral Tract Therapy**	Yes ☐<br>No ☐<br>**If NO**, please move to	0-6 months ☐<br>7-11 months ☐<br>1-3 years ☐<br>4-8 years ☐	One-off intervention ☐<br>Time limited programme ☐	Physical difficulties ☐<br>Non-physical difficulties ☐<br>Both ☐	Home ☐<br>Nursery / playgroup ☐<br>School ☐<br>Hospital ☐	Yes ☐<br>No ☐<br>Sometimes ☐	0-3 months ☐<br>4-6 months ☐<br>7-9 months ☐	Parents: Yes ☐<br>No ☐<br>School / Nursery: Yes ☐<br>No ☐

	question 31 below	9 years and above ☐	Ongoing support ☐		Other: ___________	(please state when it is effective): ___________ ___________ ___________	10-12 months ☐<br>Over 1 year ☐	
**Intervention for eating, drinking or swallowing difficulties**	**Do you use it?**	**What age range(s) do you use this with? (tick all that apply)**	**Do you provide ongoing support with this intervention? (tick one)**	**Which clinical group(s) do you use this with? (tick one)**	**Where is it delivered? (tick all that apply)**	**In your opinion, is it effective? (tick one)**	**Over what timescale does change usually occur? (tick one)**	**Is training given to parents or school / nursery? (tick Y/N for each)**
**31. Being aware of the impact of <u>movement difficulties</u> on eating and drinking**	Yes ☐<br>No ☐<br>**If NO**, please move to question 32 below	0-6 months ☐<br>7-11 months ☐<br>1-3 years ☐<br>4-8 years ☐<br>9 years and above ☐	One-off intervention ☐<br>Time limited programme ☐<br>Ongoing support ☐	Physical difficulties ☐<br>Non-physical difficulties ☐<br>Both ☐	Home ☐<br>Nursery / playgroup ☐<br>School ☐<br>Hospital ☐<br>Other: ___________	Yes ☐<br>No ☐<br>Sometimes ☐<br>(please state when it is effective): ___________ ___________ ___________	0-3 months ☐<br>4-6 months ☐<br>7-9 months ☐<br>10-12 months ☐<br>Over 1 year ☐	Parents: Yes ☐ No ☐<br>School / Nursery: Yes ☐ No ☐
**32. Being aware of the impact of <u>sensory difficulties</u> on**	Yes ☐<br>No ☐<br>**If NO**, please move to	0-6 months ☐<br>7-11 months ☐<br>1-3 years ☐<br>4-8 years ☐	One-off intervention ☐<br>Time limited programme ☐	Physical difficulties ☐<br>Non-physical difficulties ☐<br>Both ☐	Home ☐<br>Nursery / playgroup ☐<br>School ☐<br>Hospital ☐	Yes ☐<br>No ☐<br>Sometimes ☐	0-3 months ☐<br>4-6 months ☐<br>7-9 months ☐	Parents: Yes ☐ No ☐<br>School / Nursery: Yes ☐ No ☐

**eating and drinking**	question 33 below	9 years and above ☐	Ongoing support ☐		Other: ____________	(please state when it is effective): ____________ ____________ ____________	10-12 months ☐<br>Over 1 year ☐	

Intervention for eating, drinking or swallowing difficulties	Do you use it?	What age range(s) do you use this with? (tick all that apply)	Do you provide ongoing support with this intervention? (tick one)	Which clinical group(s) do you use this with? (tick one)	Where is it delivered? (tick all that apply)	In your opinion, is it effective? (tick one)	Over what timescale does change usually occur? (tick one)	Is training given to parents or school / nursery? (tick Y/N for each)
**33. Any type of strategy or programme aimed at changing behaviour at mealtimes e.g. Positive Behaviour Support, reward charts**	Yes ☐<br>No ☐<br>**If NO**, please move to question 34 below	0-6 months ☐<br>7-11 months ☐<br>1-3 years ☐<br>4-8 years ☐<br>9 years and above ☐	One-off intervention ☐<br>Time limited programme ☐<br>Ongoing support ☐	Physical difficulties ☐<br>Non-physical difficulties ☐<br>Both ☐	Home ☐<br>Nursery / playgroup ☐<br>School ☐<br>Hospital ☐<br>Other: ____________	Yes ☐<br>No ☐<br>Sometimes ☐<br>(please state when it is effective): ____________ ____________ ____________	0-3 months ☐<br>4-6 months ☐<br>7-9 months ☐<br>10-12 months ☐<br>Over 1 year ☐	Parents: Yes ☐<br>No ☐<br>School / Nursery: Yes ☐<br>No ☐
**34. Any type of visual support**	Yes ☐<br>No ☐	0-6 months ☐<br>7-11 months ☐	One-off intervention ☐	Physical difficulties ☐	Home ☐	Yes ☐<br>No ☐	0-3 months ☐	Parents: Yes ☐<br>No ☐

**e.g. visual timetable, ‘now and next’ board, ‘working towards’ board, sand-timers, social stories**	**If NO**, please move to question 35 below	1-3 years ☐<br>4-8 years ☐<br>9 years and above ☐	Time limited programme ☐<br>Ongoing support ☐	Non-physical difficulties ☐<br>Both ☐	Nursery / playgroup ☐<br>School ☐<br>Hospital ☐<br>Other: ____________	Sometimes ☐<br>(please state when it is effective):<br>____________<br>____________<br>____________	4-6 months ☐<br>7-9 months ☐<br>10-12 months ☐<br>Over 1 year ☐	School / Nursery: Yes ☐ No ☐

Intervention for eating, drinking or swallowing difficulties	Do you use it?	What age range(s) do you use this with? (tick all that apply)	Do you provide ongoing support with this intervention? (tick one)	Which clinical group(s) do you use this with? (tick one)	Where is it delivered? (tick all that apply)	In your opinion, is it effective? (tick one)	Over what timescale does change usually occur? (tick one)	Is training given to parents or school / nursery? (tick Y/N for each)
**35. Any type of modelling e.g. learning to self-feed or to accept new foods, video modelling**	Yes ☐<br>No ☐<br>**If NO**, please move to question 36 below	0-6 months ☐<br>7-11 months ☐<br>1-3 years ☐<br>4-8 years ☐<br>9 years and above ☐	One-off intervention ☐<br>Time limited programme ☐<br>Ongoing support ☐	Physical difficulties ☐<br>Non-physical difficulties ☐<br>Both ☐	Home ☐<br>Nursery / playgroup ☐<br>School ☐<br>Hospital ☐<br>Other: ____________	Yes ☐<br>No ☐<br>Sometimes ☐<br>(please state when it is effective):<br>____________<br>____________<br>____________	0-3 months ☐<br>4-6 months ☐<br>7-9 months ☐<br>10-12 months ☐<br>Over 1 year ☐	Parents: Yes ☐ No ☐<br>School / Nursery: Yes ☐ No ☐

36. Are there other interventions **you use** that are not listed?	Yes ☐ No ☐
36a. If YES, please tell us what other interventions you use<br><br>(Please list up to 3 interventions)	1.________________________________<br>________________________________<br>2.________________________________<br>________________________________<br>3.________________________________<br>________________________________
37. Are there other interventions **you don't currently use** but would like to recommend?	Yes ☐ No ☐
37a.If YES, please list up to 3 interventions you don't use but would like to recommend	Intervention 1:________________________<br>Why do you not use this now?<br>________________________________<br>________________________________<br>What appeals to you about this intervention?<br>________________________________<br>________________________________<br><br>Intervention 2:________________________<br>Why do you not use this now?<br>________________________________<br>________________________________<br><br>What appeals to you about this intervention?<br>________________________________

	____<br><br>Intervention 3:_______________________________<br><br>Why do you not use this now?<br><br>____<br><br>____<br><br>What appeals to you about this intervention?<br><br>____<br><br>____
38. What do you think are the potential benefits of interventions for eating and drinking difficulties?<br><br>(please tick all that apply)	<u>Child physical health:</u><br><br>Better general health (e.g. less colds or infections) ☐<br><br>Better sitting ☐<br><br>Fewer abnormal or unusual movements ☐<br><br>Fewer / shorter hospital admissions ☐<br><br>Improved nutrition ☐<br><br>Increased growth ☐<br><br>Less pain ☐<br><br>Weight gain ☐<br><br><u>Eating, drinking and swallowing performance:</u><br><br>Better co-ordination of swallowing and breathing ☐<br><br>Better oral-motor function (e.g. chewing, biting) ☐<br><br>Fewer breathing changes (rate, noise, effort, coughing, antibiotics courses, chest infections) linked to eating, drinking and swallowing ☐<br><br>Less aversion / avoidance of particular foods ☐<br><br>Less drooling ☐<br><br>Less food / drink spilled from lips ☐<br><br>More food / drink consumed ☐

☐

	Shorter mealtimes<br><br>Wider range of foods eaten ☐<br><br><u>Child's quality of life and participation:</u><br><br>Better mealtime one to one interaction with child ☐<br><br>Better quality of life for child ☐<br><br>Better self-feeding / independence skills ☐<br><br>Child able to communicate better e.g. to express preferences or make choices ☐<br><br>Child enjoys mealtimes more ☐<br><br>Child less frustrated or distressed at mealtimes ☐<br><br>More involvement in family's activities e.g. eating with family or outside of the home ☐<br><br><u>Parent / carer / family related outcomes:</u><br><br>Being able to eat a meal somewhere outside the home ☐<br><br>Better understanding of child's difficulties and strategies to support them ☐<br><br>Less food waste / reduced cost of food<br><br>Less parental / carer stress ☐<br><br>More opportunity to talk to others about feelings about child's eating and drinking difficulties ☐<br><br>Not having to prepare separate meals for the child ☐<br><br>Parent / carer / family enjoys mealtimes more ☐<br><br>Parent / carer / family less frustrated or distressed at mealtimes ☐<br><br>Other (please specify)<br><br>____<br><br>____
39. From the list above, which do you think are the **most important**	• ______________________________________

outcomes for the child, parents and family?<br><br>(please write 5 in the spaces provided)	• __________<br>• __________<br>• __________<br>• __________
40. Do you measure eating and drinking outcomes?	Usually ☐<br>Sometimes ☐<br>Never ☐
40a-q. If YES:<br><br>i) Which tools do you use to measure outcomes<br><br>ii) Which clinical groups do you use each tool with?<br><br>(please tick all that apply)	**a. Brief Assessment of Motor Function - Oral Motor Deglutition Scale (BAMF-OMD):**<br><br>i) Do you use this tool?<br><br>Yes ☐<br>No ☐<br><br>ii) If YES, which clinical groups do you use it with?<br><br>Physical disabilities ☐ Non-physical disabilities ☐<br>8 years and under ☐ Over 8 years ☐<br><br>**b. Behavioural Assessment Scale of Oral Functions in Feeding (BASOFF):**<br><br>i) Do you use this tool?<br><br>Yes ☐<br>No ☐<br><br>ii) If YES, which clinical groups do you use it with?<br><br>Physical disabilities ☐ Non-physical disabilities ☐<br>8 years and under ☐ Over 8 years ☐<br><br>**c. Dysphagia Disorders Survey (DDS):**<br><br>i) Do you use this tool?

	Yes ☐<br>No ☐<br>ii) If YES, which clinical groups do you use it with?<br>Physical disabilities ☐ Non-physical disabilities ☐<br>8 years and under ☐ Over 8 years ☐<br><br>**d. Feeding Behaviour Scale (FBS):**<br>i) Do you use this tool?<br>Yes ☐<br>No ☐<br>ii) If YES, which clinical groups do you use it with?<br>Physical disabilities ☐ Non-physical disabilities ☐<br>8 years and under ☐ Over 8 years ☐<br><br>**e. Functional Feeding Assessment (FFA):**<br>i) Do you use this tool?<br>Yes ☐<br>No ☐<br>ii) If YES, which clinical groups do you use it with?<br>Physical disabilities ☐ Non-physical disabilities ☐<br>8 years and under ☐ Over 8 years ☐<br><br>**f. Gisel Video Assessment (GVA):**<br>i) Do you use this tool?<br>Yes ☐<br>No ☐<br>ii) If YES, which clinical groups do you use it with?<br>Physical disabilities ☐ Non-physical disabilities ☐

	8 years and under ☐ Over 8 years ☐<br><br>**g. Oral Motor Assessment Scale (OMAS):**<br><br>i) Do you use this tool?<br><br>Yes ☐<br><br>No ☐<br><br>ii) If YES, which clinical groups do you use it with?<br><br>Physical disabilities ☐ Non-physical disabilities ☐<br><br>8 years and under ☐ Over 8 years ☐<br><br>**h. Pre-Speech Assessment Scale (PSAS):**<br><br>i) Do you use this tool?<br><br>Yes ☐<br><br>No ☐<br><br>ii) If YES, which clinical groups do you use it with?<br><br>Physical disabilities ☐ Non-physical disabilities ☐<br><br>8 years and ☐ ☐<br><br>**i. Schedule for Oral Motor Assessment (SOMA):**<br><br>i) Do you use this tool?<br><br>Yes ☐<br><br>No ☐<br><br>ii) If YES, which clinical groups do you use it with?<br><br>Physical disabilities ☐ Non-physical disabilities ☐<br><br>8 years and under ☐ Over 8 years ☐<br><br>**j. Parenting Stress Index (PSI):**<br><br>i) Do you use this tool?

	Yes ☐<br>No ☐<br>ii) If YES, which clinical groups do you use it with?<br>Physical disabilities ☐ Non-physical disabilities ☐<br>8 years and under ☐ Over 8 years ☐<br><br>**k. Parental Stress Scale (PSS):**<br>i) Do you use this tool?<br>Yes ☐<br>No ☐<br>ii) If YES, which clinical groups do you use it with?<br>Physical disabilities ☐ Non-physical disabilities ☐<br>8 years and under ☐ Over 8 years ☐<br><br>**l. Autism Parenting Stress Index (APSI):**<br>i) Do you use this tool?<br>Yes ☐<br>No ☐<br>ii) If YES, which clinical groups do you use it with?<br>Physical disabilities Non-physical disabilities ☐<br>8 years and under ☐ Over 8 years ☐<br><br>**m. Parent Coping Scale (PCS):**<br>i) Do you use this tool?<br>Yes ☐<br>No ☐<br>ii) If YES, which clinical groups do you use it with?<br>Physical disabilities ☐ Non-physical disabilities ☐

	8 years and under ☐ Over 8 years ☐<br><br>**n. Questionnaire on Resources and Stress (QRS-F):**<br><br>i) Do you use this tool?<br><br>Yes ☐<br><br>No ☐<br><br>ii) If YES, which clinical groups do you use it with?<br><br>Physical disabilities ☐ Non-physical disabilities ☐<br><br>8 years and under ☐ Over 8 years ☐<br><br>**o. Vineland Adaptive Behaviour Scales (VABS):**<br><br>i) Do you use this tool?<br><br>Yes ☐<br><br>No ☐<br><br>ii) If YES, which clinical groups do you use it with?<br><br>Physical disabilities ☐ Non-physical disabilities ☐<br><br>8 years and under ☐ Over 8 years ☐<br><br>**p. Family Impact Questionnaire (FIQ):**<br><br>i) Do you use this tool?<br><br>Yes ☐<br><br>No ☐<br><br>ii) If YES, which clinical groups do you use it with?<br><br>Physical disabilities ☐ Non-physical disabilities ☐<br><br>8 years and under ☐ Over 8 years ☐<br><br>**q. Therapy Outcome Measures (TOMS):**

	i) Do you use this tool?<br>Yes ☐<br>No ☐<br>ii) If YES, which clinical groups do you use it with?<br>Physical disabilities ☐ Non-physical disabilities ☐<br>8 years and under ☐ Over 8 years ☐
41. Do **you use** any other outcome measures not listed above?	Yes ☐<br>No ☐
41a. If YES, please:<br>i) List up to 3 outcome measures you use<br>ii) State which clinical groups you use them with	1. Name of tool:________________________________<br>Physical disabilities ☐ Non-physical disabilities ☐<br>8 years and under ☐ Over 8 years ☐<br>2. Name of tool:________________________________<br>Physical disabilities ☐ Non-physical disabilities ☐<br>8 years and under ☐ Over 8 years ☐<br>3. Name of tool:________________________________<br>Physical disabilities ☐ Non-physical disabilities ☐<br>8 years and under ☐ Over 8 years ☐
42. Are there other measurement tools **you would like to use**?	Yes ☐<br>No ☐
42a and b. If YES, please list up to 3 other tools you would like to use.	Measurement tools:<br>1.____<br>____

	2.____<br>____<br>3.____<br>____<br><br>Why do you not use them now?<br>1.____<br>____<br>2.____<br>____<br>3.____<br>____

Please continue onto the next page.

This is the end of the first stage of the research. <u>We hope that you will agree to also take part in the second stage of the research</u>. This will involve completing another survey to help identify which interventions for eating, drinking and swallowing difficulties might be suitable for future research trials.

43. Please tick if you would like to:

Go into a prize draw to win one of five £100 vouchers	☐
Receive a summary of the survey results	☐
Complete another survey in a few months	☐

43a. If you have ticked a box above, please give your contact details so that we may contact you in the future:

Name:

Email address:

Postal address:

Thank you for taking part in this survey. We will summarise the results of the survey and the wider study in written summaries and on the FEEDS website: http://research.ncl.ac.uk/neurodisability/ourstudies/feedsreview

Please contact us if you have any questions using the contact details below:

Dr Helen Taylor
FEEDS study
Development & Disability Group, Newcastle University
3rd Floor, Sir James Spence Institute
Royal Victoria Infirmary
Queen Victoria Road
Newcastle upon Tyne
NE1 4LP

Tel: +44 (0)191 282 1379 Email: Feeds@ncl.ac.uk
