# Supplementary material for: Informing creation of the FEEDS Toolkit to support parent-delivered interventions for eating, drinking and swallowing difficulties in young children with neurodisability: intervention use by neurodevelopmental diagnosis and healthcare professional role
Source: BMJ Paediatr Open. 2024 Aug 24;8(1):e002394. doi: 10.1136/bmjpo-2023-002394 (PMC11733779; doi:10.1136/bmjpo-2023-002394)
Supplement: online supplemental file 2 [file bmjpo-8-1-s002.pdf]

Supplementary Material 2: Interventions presented in the survey

Intervention	Description
Positioning	Ensuring a child has the best posture to eat and drink food safely and efficiently
Manoeuvres	Giving direct physical support to a child when eating or drinking to improve the movements needed to bite, chew and swallow
Oral-motor exercises	Exercises done with a child with the aim of improving their control of their mouth, jaw, tongue or lips
Medication	Any prescribed medicine that could affect eating and drinking
Schedule of meals	Setting the timing of mealtimes to encourage a child’s appetite and readiness to eat and drink, and establish a mealtime routine
Food or drink modification	Changing aspects of the child’s food or drink, such as the consistency, temperature, taste, amount or presentation
Modification of utensils	Using different spoons, forks, plates, cups or bottles
Modification of the environment	Changing the physical or social setting at mealtimes
Sensory aids	e.g. glasses or hearing aids
Energy supplements	Any energy or calorie supplement given orally or via feeding tube
Training to wait for a child’s cues for feeding	Helping parents/caregivers to recognise the signs that a child is ready to take another mouthful of food or drink

Pacing of food at mealtimes	Changing the speed at which each mouthful of food or drink is taken by a child
Enhancing child/feeder communication strategies at mealtimes	Improving interaction between a child and the person feeding them during mealtimes
Modifying social eating and drinking opportunities	Any change in the physical environment or behaviour of others when children eat or drink in company
Hand over hand prompting	Physically guiding the child’s hand to encourage them to grasp or move food / equipment
Counselling	Collaboration between professional and parent to enhance understanding of causes, extent and impacts of feeding difficulties and promote parents’ wellbeing
Desensitisation programme for food avoidance	Activities aimed at gradually introducing a child to new or previously rejected foods and drinks
Desensitisation programme for oral sensations	Activities aimed at reducing a child’s adverse reactions to different sensory experiences linked to eating and drinking
Sensory stimulation	Touch-based stimulation on and around the lips and mouth in an attempt to reduce sensory based feeding difficulties
Sensorimotor therapies	Interventions focusing on both sensation (vision, hearing, smell, taste, touch, and proprioception) and motor output for learning new skills.
Sharing information on the impact of sensory difficulties on eating and drinking	

Sharing information on the impact of movement difficulties on eating and drinking	
Strategies/programmes aimed at changing behaviour at mealtimes	Strategies to encourage a child to behave appropriately at mealtimes
Visual supports	Use of pictures, a ‘countdown clock’, or social stories to increase a child’s understanding of what happens during mealtimes
Modelling	Giving a child the opportunity to learn from others by eating and drinking with them
