# Supplementary material for: Informing creation of the FEEDS Toolkit to support parent-delivered interventions for eating, drinking and swallowing difficulties in young children with neurodisability: intervention use by neurodevelopmental diagnosis and healthcare professional role
Source: BMJ Paediatr Open. 2024 Aug 24;8(1):e002394. doi: 10.1136/bmjpo-2023-002394 (PMC11733779; doi:10.1136/bmjpo-2023-002394)
Supplement: Supplementary file 3 [file bmjpo-2023-002394.reviewer_comments.pdf]

PEER REVIEW HISTORY

BMJ Paediatrics Open publishes all reviews undertaken for accepted manuscripts. Reviewers are asked to complete a checklist review form and are provided with free text boxes to elaborate on their assessment. These free text comments are reproduced below.

ARTICLE DETAILS

**TITLE (PROVISIONAL)**	Informing creation of the FEEDS Toolkit to support parent-delivered interventions for eating, drinking, and swallowing difficulties in young children with neurodisability: intervention use by neurodevelopmental diagnosis and healthcare professional role
**AUTHORS**	Shaw, Emogene<br>Pennington, Lindsay<br>Andrew, Morag<br>Taylor, Helen<br>Cadwgan, Jill<br>Sellers, Diane<br>Morris, Christopher<br>Garland, Deborah<br>Parr, Jeremy

VERSION 1 – REVIEW

**REVIEWER**	Mrs. Rebecca Davidson<br>NHS Lanarkshire<br>Speech and Language Therapy<br>Coathill Hospital<br>Glen Doe Building<br>Coatbridge<br>Lanarkshire<br>ML5 4DN<br>United Kingdom of Great Britain and Northern Ireland
**REVIEW RETURNED**	06-Dec-2023

**GENERAL COMMENTS**	This survey, whilst a welcome addition to research in this area, remains skewed towards children with an ASD diagnosis. It is disappointing as ever that not all four nations appear well presented in survey returns and fathers remain under-represented (this is probably not uncommon in research). Some interventions appear surprisingly low eg counselling which suggests it perhaps isn't widely recognised by parents/carers as such. Another disappointing finding would be the 0% finding of using visual supports in children with cerebral palsy, which is perhaps the result of the low number of children with cerebral palsy represented in this study. I wonder whether there is an opportunity here for the interventions with low use and low evidence base to be considered to be removed from the FEEDS toolkit? There is a risk that some interventions could be used as 'usual practice' without the consideration of 'best practice'. The Toolkit is going to be a welcome addition to the work of clinicians from a range of professionals working within the field of paediatric neurodisability and eating, drinking and swallowing difficulties.

**REVIEWER**	Dr. Catherine Tuffrey

	United Kingdom of Great Britain and Northern Ireland
**REVIEW RETURNED**	02-Mar-2024

**GENERAL COMMENTS**	This is an important topic and the FEEDS study in its entirety has produced useful findings.<br>I found this paper rather confusing to read. It is not very easy to get one's head around which part of the larger study this data appertains to.Some sort of flow chart may have helped. I was not clear why it was being reported separately.<br>The finding that the child's primary diagnosis does not necessarily dictate the interventions used is not a surprise as many children will have more than one diagnosis, and the particular eating difficulties a child has are related to their activitiy limitations and other medical impairment/experiences rather than the underlying medical cause. For example, many children with CP and DS have autistic type features in addition to other difficulties. Any child who has had to receive NGT feeds for whatever reason, may then show aversive behaviours. And any child (many without disabilities) may benefit from what may be regarded as basic parenting in terms of appropriate timing of meals.<br>What I am not clear we know from this study, is whether interventions are being used appropriately and also whether an umbrella description, for example of 'positioning' - would reflect the same intervention in a child with ASD vs one with CP. So whilst saying a range of interventions may be recommended, I am not sure this study tells us that this range is also appropriate, or really what is involved.<br>I could not tell from the paper, how the data had affected the toolkit design other than telling people that a range of interventions may be used, which I would think to be self-evident.<br>In the 'What this study adds' I would query that the second point is really new information.The wording of the 3rd point I found confusing. In addition, I would have thought that the work to establish the evidence base would be more appropriate in advising clinicians on choice of intervention.<br>No comment is made about the relatively low representation of non-white ethnicities in the parent carer respondents.<br>I wonder whether the tables of results would be easier for the reader to see if presented graphically rather than in tables. There is a lot of data which is hard for the reader to assimilate.<br>I wonder whether part of the reason that professionals recommend interventions not usually associated with their usual professional focus reflects the fact that they are indicating that they would make recommendations as a team rather than as individuals.

VERSION 1 – AUTHOR RESPONSE

Reviewer 1:

We thank reviewer 1 for their positive feedback regarding the FEEDS Toolkit as a welcome addition to the work of clinicians from a range of professionals working within the field of paediatric neurodisability and eating, drinking and swallowing difficulties.

1. This survey, whilst a welcome addition to research in this area, remains skewed towards children with an ASD diagnosis. It is disappointing as ever that not all four nations appear well

presented in survey returns and fathers remain under-represented (this is probably not uncommon in research).

Response: We agree with reviewer 1 that the population included is not representative of all families, and that all four nations are not well represented. Our survey was promoted via a variety of methods seeking broad range of children across UK; nevertheless we suggest the findings are valuable despite these limitations. We have added these limitations to the discussion (page 13, lines 3-8 and 11-14).

Changes within the text:
'Despite using multiple methods to distribute the survey (5), respondents were mostly mothers, and most were white. Representation across the four nations was more limited than we hoped/expected. Future data collected via the FEEDS Toolkit directly will allow for the collection of more representative data'

2. Some interventions appear surprisingly low eg counselling which suggests it perhaps isn't widely recognised by parents/carers as such. Another disappointing finding would be the 0% finding of using visual supports in children with cerebral palsy, which is perhaps the result of the low number of children with cerebral palsy represented in this study

Response: Intervention definitions were provided to parent carers and healthcare professionals completing the survey (which we have now included as supplementary information and discussed in our methods- page 13 lines 7-11), however parent carers and healthcare professionals may have different expectations on 'counselling', and indeed the other interventions, and this will be considered for the toolkit content to ensure clear wording/ definitions. We agree that it is likely the use of visual support in cerebral palsy has been affected by the lower number of cerebral palsy responses, and this finding will be interesting to investigate further when able to analyse intervention use via data directly taken from the toolkit.

Changes within the text:
'These limitations may have resulted in low reported use of some interventions (for example, no reported use of visual supports for children with CP). In the future, data collected through studies of FEEDS Toolkit use will be used to understand which interventions are used by parents of children with a range of diagnoses.'

3. I wonder whether there is an opportunity here for the interventions with low use and low evidence base to be considered to be removed from the FEEDS toolkit? There is a risk that some interventions could be used as 'usual practice' without the consideration of 'best practice'.

Response: The goal is for the FEEDS toolkit to facilitate shared decision making between parent carers and healthcare professionals. This survey highlights current interventions used across the country and results support the suitability of the toolkit for integration into current practice. It is hoped that the toolkit, by facilitating shared decision making will lead to better feeding outcomes for children. Retaining the interventions that are used less frequently will allow the whole range of interventions options to be discussed by teams supporting children. We have added further clarification (page 4, lines 24-26 and lines 32-33) to make this clearer to readers.

Changes within the text:
'Subsequently practitioners asked about intervention use by HP type and by condition. We conducted analyses in response to practitioner questions and to support development of the FEEDS Toolkit.'
'The findings from this study aimed to further analyse the national survey responses from the FEEDS review to inform on current intervention practices within the NHS, and determine whether intervention use varied by children's main diagnosis and whether intervention recommendations varied by HP role (5).'

Reviewer 2:

We thank reviewer 2 for their comments on our paper. We have addressed their comments below:

1. I wonder whether the tables of results would be easier for the reader to see if presented graphically rather than in tables. There is a lot of data which is hard for the reader to assimilate.

Response: We thank reviewer 2 for this suggestion. The creation of graphs was previously done by the research team and have been added to the end of this letter in the attached document. However, the graphs do not make the results clearer. We consider that the tables provide the detailed information more transparently and allow readers to fully understand the results.

2. No comment is made about the relatively low representation of non-white ethnicities in the parent carer respondents.

Response: We have added this to our discussion (page 13, lines 11-12).

Changes to the text:
'Finally, despite using multiple methods to distribute the survey (5), respondents were mostly mothers, and most were white.'

3. It is not very easy to get one's head around which part of the larger study this data appertains to. Some sort of flow chart may have helped. I was not clear why it was being reported separately.

Response: Following on from the original paper published regarding the FEEDS survey, the question was posed to the study team about intervention use by HP type and condition and thus this analysis has been conducted. We appreciate reviewer 2's feedback and have included more information to ensure readers understand how this survey originally fit within the larger FEEDS review (page 4, lines 14-17) and to make it clearer that this is new data and has not been published previously (page 4, lines 24-26).

Changes to the text:
'The FEEDS national survey aimed to 'to establish current UK clinical practice in relation to the interventions recommended and used for EDSD in young children with neurodisability and the outcomes measured, from the perspective of parents and health and education professionals' (5).'

‘Subsequently practitioners asked about intervention use by HP type and by condition. We conducted analyses in response to practitioner questions and to support development of the FEEDS Toolkit.’

4. The finding that the child's primary diagnosis does not necessarily dictate the interventions used is not a surprise as many children will have more than one diagnosis, and the particular eating difficulties a child has are related to their activity limitations and other medical impairment/experiences rather than the underlying medical cause. For example, many children with CP and DS have autistic type features in addition to other difficulties. Any child who has had to receive NGT feeds for whatever reason, may then show aversive behaviours. And any child (many without disabilities) may benefit from what may be regarded as basic parenting in terms of appropriate timing of meals.

Response: We agree with reviewer 2 that often children with a neurodisability have more than one diagnosis, or features consistent with other diagnoses, which may impact their feeding, and this has been addressed in our discussion (page 13, lines 4-7). It is reassuring that the results agree with what clinicians would expect; showing this in an evidence-based way is important. These findings underpin plans for the Toolkit to be individualised and not focus on specific diagnoses.

Referenced text:
‘the presence of co-morbidities, co-existing diagnoses and their influence on feeding could not be considered, as only information about main neurodevelopmental diagnosis was collected’

5. What I am not clear we know from this study, is whether interventions are being used appropriately and also whether an umbrella description, for example of 'positioning' - would reflect the same intervention in a child with ASD vs one with CP. So whilst saying a range of interventions may be recommended, I am not sure this study tells us that this range is also appropriate, or really what is involved.

Response: Determining whether interventions are currently being used appropriately was not an aim of the study. Nonetheless we agree this is important to determine and we hope that future data collected utilising the FEEDS Toolkit may be able to help answer this question. We agree that a positioning intervention would be very different for a child with CP in comparison to a child with ASD and this is explained within the toolkit. This paper has informed us and the community about on current intervention use and, with the addition of other aspects of the larger FEEDS study, informed the creation of the FEEDS toolkit.

6. I could not tell from the paper, how the data had affected the toolkit design other than telling people that a range of interventions may be used, which I would think to be self-evident.

Response: We thank reviewer 2 for this feedback and have subsequently changed the text within the paper to explain the aim of this paper more clearly and how this data has benefited Toolkit creation (page 12, lines 4-7). As discussed in 4, this data reflects the individualised approach required for each child, and therefore the benefit of a toolkit such as the FEEDS toolkit which will inform parents and the MDT of interventions and aid shared decision making,

Changes in the text:

‘The results provide information about current UK feeding services and their intervention use. The results have also been used to develop the FEEDS Toolkit, ensuring it is relevant to a wide range of healthcare professionals, and to parents of children with a range of diagnoses.’

7. In the 'What this study adds' I would query that the second point is really new information. The wording of the 3rd point I found confusing. In addition, I would have thought that the work to establish the evidence base would be more appropriate in advising clinicians on choice of intervention.

Response: We have modified text within this area to clearly demonstrate to readers the implications for the data presented (as described above) in response to the associate editor.

8. I wonder whether part of the reason that professionals recommend interventions not usually associated with their usual professional focus reflects the fact that they are indicating that they would make recommendations as a team rather than as individuals.

Response: Thank you. This is an important consideration. Within the healthcare professional and parent carer surveys, participants were introduced to the interventions and asked ‘Do you use it?’. The interventions also included examples to improve understanding of what each intervention involved. The questionnaire has been included in the supplementary information (Supplementary Material 1) for readers to review. The FEEDS toolkit aims to increase awareness on the breadth of interventions which can be utilised, and will encouraged discussions across the MDT and with parent carers regarding intervention choice.

VERSION 2 – REVIEW

**REVIEWER**	Mrs. Rebecca Davidson<br>NHS Lanarkshire<br>Speech and Language Therapy<br>Coathill Hospital<br>Glen Doe Building<br>Coatbridge<br>Lanarkshire<br>ML5 4DN<br>United Kingdom of Great Britain and Northern Ireland
**REVIEW RETURNED**	30-May-2024

**GENERAL COMMENTS**	This is an excellent paper which addresses the realities and practicalities of multi-agency working with young children with neurodisability and feeding difficulties.
